# Prevalence of Nocturnal Enuresis Among Children and Its Association With the Mental Health of Mothers in Northern Saudi Arabia

**DOI:** 10.7759/cureus.22232

**Published:** 2022-02-15

**Authors:** Anfal Nayir H Alanazi, Reem Salem M Alanazi, Eman N Alanazi, Reham M Alanazi, Unaib Rabbani

**Affiliations:** 1 Family Medicine, Saudi Board of Family Medicine Joint Program, Northern Border Region, Arar, SAU; 2 Family Medicine Academy, Qassim Health Cluster, Qassim, SAU

**Keywords:** saudi arabia, mothers, stress, mental health, outcomes, nocturnal enuresis

## Abstract

Background

Nocturnal enuresis (NE) is troubling for children and their families. This study aims to investigate the prevalence of NE, its associated health problems, and the outcome of the provided management among children aged 6-18 years and to assess the impact of NE on the mental health of mothers in Northern Saudi Arabia.

Methodology

A retrospective cross-sectional study was conducted in Arar, Northern Saudi Arabia, among children aged 6-18 years old. Data were collected using a structured questionnaire including a Perceived Stress Scale (PSS). Means ± standard deviations (SDs) were used to represent quantitative data, and frequencies and percentages were used to represent qualitative data. Ordinal logistic regression was used to assess the association of NE with perceived stress.

Results

A total of 420 participants were included in this study. Nocturnal enuresis was reported in 24% of the respondents’ children. Around 51% of the mothers know about the causes of NE. Nocturnal enuresis caused embarrassment and social shame to 71% of the mothers. Two-thirds (66%) of the mothers wake up the child at night for urination. Three-quarters (76%) of the participants reported improvement on decreasing fluid intake before sleeping. Of the mothers, 19% perceived low stress, 78% perceived medium stress, and 3% perceived high stress. NE was associated with a higher risk of stress (adjusted odds ratio (OR): 2.14; 95% confidence interval (CI): 1.05-4.37).

Conclusion

About a quarter of the children suffer from NE, of which a large proportion of mothers face embarrassment and shame. There was a significant association between NE and a higher level of stress. Mothers of children with NE should be provided with counseling and social support to ensure good mental health.

## Introduction

Nocturnal enuresis (NE), also referred to as nighttime incontinence, is defined as the repeated, spontaneous passage of urine during sleep beyond the age of anticipated full nocturnal bladder control, which is generally accepted to be five years of age [1]. It is three times more common than daytime incontinence, and it affects 6.7% of younger children and 2.8% of older children [2]. Meanwhile, the incidence among adolescents aged 15-18 is 1% [3]. It is three times more common among young boys than young girls [4]. Several theories have been proposed for the etiology of nocturnal enuresis. These include genetic and familial factors, psychological factors, bladder diseases, hormonal dysregulation, and sleep disorders [5]. Genetic predisposition seems to be the most supported associated variable. The review by Nørgaard et al. showed that when the two parents of the child were enuretic as children, the risk of their offspring having nocturnal enuresis was as high as 77% [6].

Nocturnal enuresis is often troubling for children and their families, which puts them in stressful situations. Although studies examining primary enuresis and its association with psychological comorbidities are limited, it has been shown that nocturnal enuresis is associated with externalizing problem behaviors and attention deficit hyperactivity disorder (ADHD) [7,8]. The study by Biederman et al. found that the association between ADHD and nocturnal enuresis is of a 30% greater chance than non-enuretic adolescents [9]. On the other hand, studies found that enuresis is associated with lower self-esteem and greater levels of internalizing behaviors, which were attributed to distress, shame, and familiar intolerance of the condition [10,11].

Nocturnal enuresis has serious consequences for the child and the parents. Nocturnal enuresis can contribute to a variety of cognitive, social, and psychological problems, including embarrassment, blushing, loss of dignity, and aggressive behavior [12]. Many children with NE experience behavioral changes such as lack of self-esteem, loneliness, decreased ambition, and anxiety symptoms. These children are frequently underachievers in school and become a source of concern for their families and schools. Therefore, it is critical to identify at-risk children and take therapeutic measures [13].

Previous studies in Saudi Arabia have shown a prevalence of NE in different regions of the kingdom ranging from 7.8% to 92.8% [14-18]. A history of pinworm infestation, lack of breastfeeding, poor academic performance, and a lower level of father education have been associated with NE [17]. Other factors associated with NE include female gender, young age, a family history of enuresis, deep sleep, a history of urinary tract infections, and other social and psychological problems [18].

Studies outside the KSA around the world reported that the prevalence of NE ranges from 4% to 18% [19-22]. Enuresis was reported to affect the quality of life, as Shaheen et al.’s study reported that 68% of enuretic children had a moderately poor quality of life, while only 3% had a severely poor quality of life [22]. The significant predictors for poorer quality of life among the affected children were ranked as follows: medication to treat enuresis, accidental urination, moderate to severe clinical affection, consumption of caffeinated drinks, older age > 10 years, and intolerant mothers to the enuretic act [22].

To the best of our knowledge, there are no previous studies assessing the prevalence of NE and its associated factors in Arar, Northern Saudi Arabia. Hence, this study aims to investigate the prevalence of nocturnal enuresis, its associated health problems, and the outcome of the provided management among children aged 6-18 years and to assess the impact of NE on the quality of life of mothers in Arar, Northern Saudi Arabia.

## Materials and methods

Study settings and population

A cross-sectional study was conducted among mothers attending primary healthcare centers and public parks in Arar, Northern Saudi Arabia, from June to October 2021.

Inclusion criteria

We included mothers aged between 18 and 60 years with at least one child below 18 years of age.

Exclusion criteria

Those with severe mental illnesses and hearing problems were excluded.

Sample size

The sample size of this study was calculated using the WHO’s sample size determination in health studies. We assumed an expected prevalence of NE to be 50% to achieve the maximum sample size. At 95% confidence level and 5% bound on error, the required sample size was 384. We further inflated the sample by 10% for missing and incomplete information. The final sample required was 420 mothers.

Sampling

Convenience sampling was used to recruit mothers from two primary healthcare centers, one shopping mall, and one public park in Arar. The participants were invited to participate and be assessed for eligibility criteria. Consent to participate was obtained from those who met the eligibility criteria.

Data collection

Data were collected using a structured questionnaire composed of three main sections. The first section included the sociodemographic characteristics of the child and parents. The second section included variables related to the history of NE in the family, knowledge of the mothers about NE, risk factors, management, and caring about the affected child. The third section assessed the impact of NE on the quality of life of mothers. The 10-item Perceived Stress Scale (PSS-10) was used for the measurement of stress among the mothers [23]. This tool has been widely used among various populations and groups to assess stress. The Arabic translation of the PSS-10 has been done and validated in a number of studies [24,25]. In this study, we used a validated Arabic version of the PSS-10. Each item in the PSS-10 is a five-point Likert scale that measures the frequency of events/feelings. The responses are coded as follows: 0 = never, 1 = almost never, 2 = sometimes, 3 = fairly often, and 4 = very often. The total score on the PSS-10 would range from 0 to 40. The following cutoff scores have been defined: low stress, 0-13; moderate stress, 14-26; and high stress, 27-40.

Statistical analysis

Data analysis was carried out using SPSS version 23.0 (IBM Corporation, Armonk, NY, USA). Means and standard deviations (SDs) were calculated for continuous variables, while frequencies and proportions were used to summarize qualitative variables. Frequencies and proportions were also calculated for mothers reporting children with NE to estimate the prevalence of NE. The PSS-10 items were coded according to the standard procedure, including reverse coding for four items (4,5,7, and 8). The sum of scores was calculated and categorized according to the defined cutoff. The association of NE with perceived stress was assessed using ordinal logistic regression. P-value < 0.05 was considered statistically significant.

Ethical considerations

All participants provided written informed consent. The study was approved by Qassim Regional Bioethics Committee (#1443-441039).

## Results

A total of 420 mothers were included in this study. Among the youngest children of the mothers, male children were 44.5%, and the mean age of the youngest child was 8.08 ± 2.09 years. The median birth order was 2.00. Table 1 describes the sample characteristics in more detail. The majority of the children were delivered through vaginal delivery (74.8%). Most of the children’s siblings had NE (91.4%), while 6.4% of the children’s parents also suffered from NE. Regarding health characteristics, 15.5% of the participants were suffering from anemia, 4.3% had a parasitic infestation, 3.8% had diabetes mellitus (DM), 7.9% had a history of repeated or chronic urinary tract infection, 3.6% were suffering from delayed development, and 7.6% reported from psychological problems. Near half of the mothers had a university or higher level of education (47.1%), and 42.1% of the fathers had a university or higher level of education. According to family income, the majority of the participants perceived their income to be insufficient (65.5%). Regarding parental status, most of the parents were living together (95%).

**Table 1:** Sociodemographic, birth, and health characteristics of the youngest child (n = 420) IQR: interquartile range NE: nocturnal enuresis

Variables	% (n)
Age of the youngest child (years)	
Mean (SD)	8.08 (2.09)
Gender of the youngest child	
Female	233 (55.5)
Male	187 (44.5)
Gestational age	
Seven months	2 (0.5)
Eight months	18 (4.3)
Nine months	400 (95.2)
Birth order	
Median (IQR)	2.0 (1–4)
Type of delivery	
Vaginal	314 (74.8)
Cesarean section	106 (25.2)
Hospital admission after delivery	
No	321 (76.4)
Yes	99 (23.6)
Sibling suffering from NE	
No	384 (91.4)
Yes	36 (8.6)
Parents suffering from NE	
No	393 (93.6)
Yes	27 (6.4)
History of anemia	
No	355 (84.5)
Yes	65 (15.5)
History of parasitic infestation	
No	402 (95.7)
Yes	18 (4.3)
History of diabetes mellitus (DM)	
No	404 (96.2)
Yes	16 (3.8)
History of repeated or chronic urinary tract infection	
No	387 (92.1)
Yes	33 (7.9)
History of delayed development	
No	405 (96.4)
Yes	15 (3.6)
History of psychological problems	
No	388 (92.4)
Yes	32 (7.6)
Mother’s educational level	
Primary/preparatory	77 (18.3)
Secondary	145 (34.5)
University or more	198 (47.1)
Father’s educational level	
Primary/preparatory	70 (16.7)
Secondary	173 (41.2)
University or more	177 (42.1)
Number of family members	
Median (IQR)	5 (4–7)
Family income (perceived)	
Enough	101 (24.0)
Not enough	275 (65.5)
Enough and more	44 (10.5)
Parent status	
Live together	399 (95.0)
Divorced	17 (4.0)
One of them is dead	4 (1.0)

**Figure 1:**
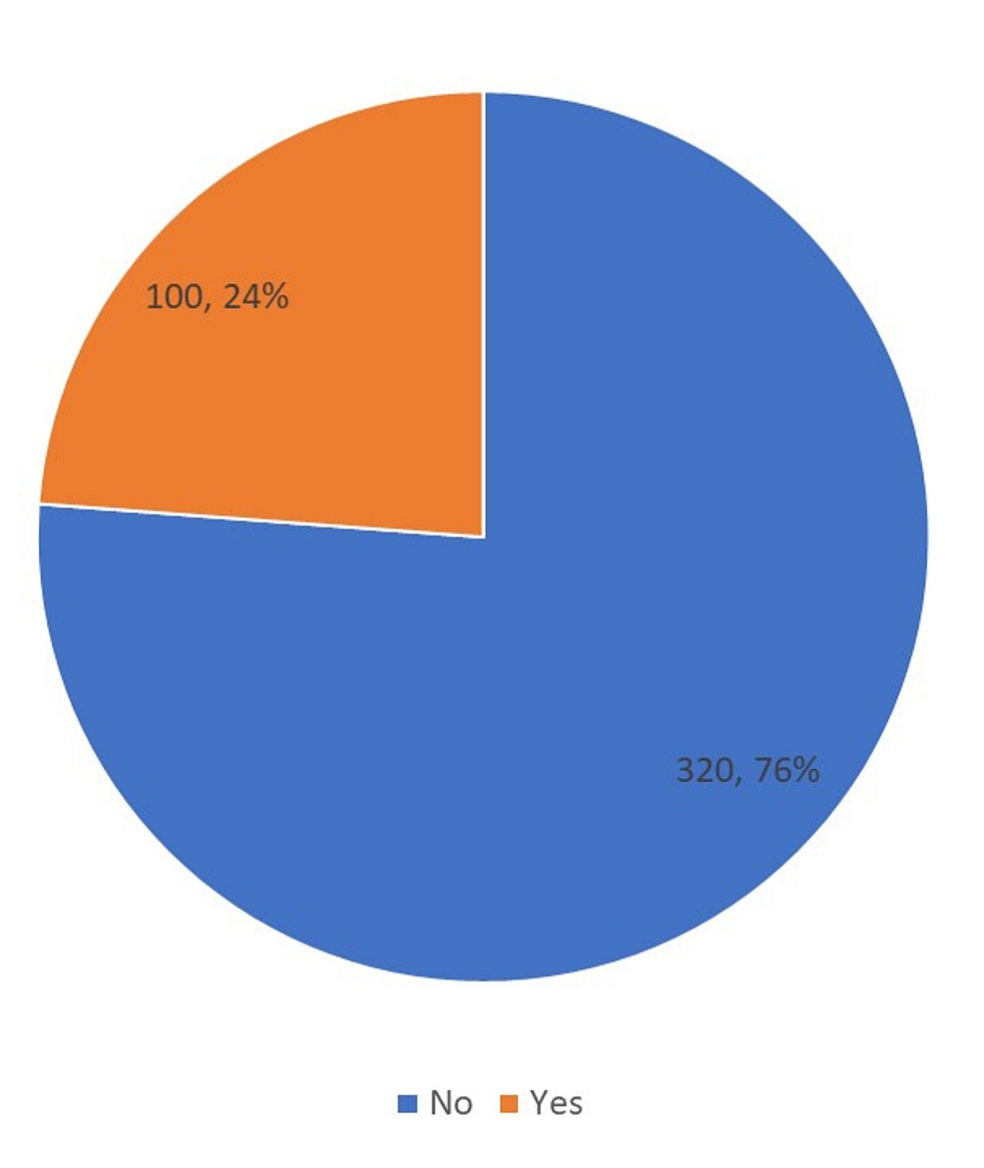
Prevalence of nocturnal enuresis among children aged 6-18 years in Arar, KSA

Nocturnal enuresis was reported in 24% of the respondents’ children (Figure 1). Among the children who have NE, most of them have the timing of enuresis at night only (89%). Around 46% of the children had 1-2 times per week of enuresis. Around 51% of the mothers know about the causes of NE. Among these causes, weakness in the muscles of the lower urinary tract, psychological problems, urinary system, and anemia were the most frequently reported causes. Nocturnal enuresis caused embarrassment and social shame to 71% of the affected children. Two-thirds (66%) of the mothers would wake up their child at night to urinate. Three-quarters (76%) of the participants reported improvement on decreasing fluid intake before sleeping (Table 2).

**Table 2:** Mothers’ knowledge and practices, and characteristics of nocturnal enuresis (n = 100)

Variables	% (n)
Mother knows about the causes of nocturnal enuresis	
Yes	51 (51.0)
No	49 (49.0)
Causes of nocturnal enuresis (n = 95)	
Weakness in the muscles of the lower urinary tract	22 (23.2)
Problems or damage of the urinary tract or nerves that control the urinary system	4 (4.2)
Psychological problems	18 (18.9)
Urinary tract infections	20 (21.1)
Hereditary	11 (11.6)
Anemia	3 (3.2)
Irritability	17 (17.9)
Timing of enuresis	
Night only	89 (89.0)
Day and night	11 (11.0)
Frequency per week	
1–2	46 (46.0)
3–4	39 (39.0)
5–7	15 (15.0)
Improvement on decreasing fluid intake before sleeping	
No	24 (24.0)
Yes	76 (76.0)
Mother keen to wake the child at night to urinate	
No	34 (34.0)
Yes	66 (66.0)
The problem cause embarrassment and social shame to the affected child	
No	29 (29.0)
Yes	71 (71.0)
Sought medical advice	
No	36 (36.0)
Yes	64 (64.0)

**Figure 2:**
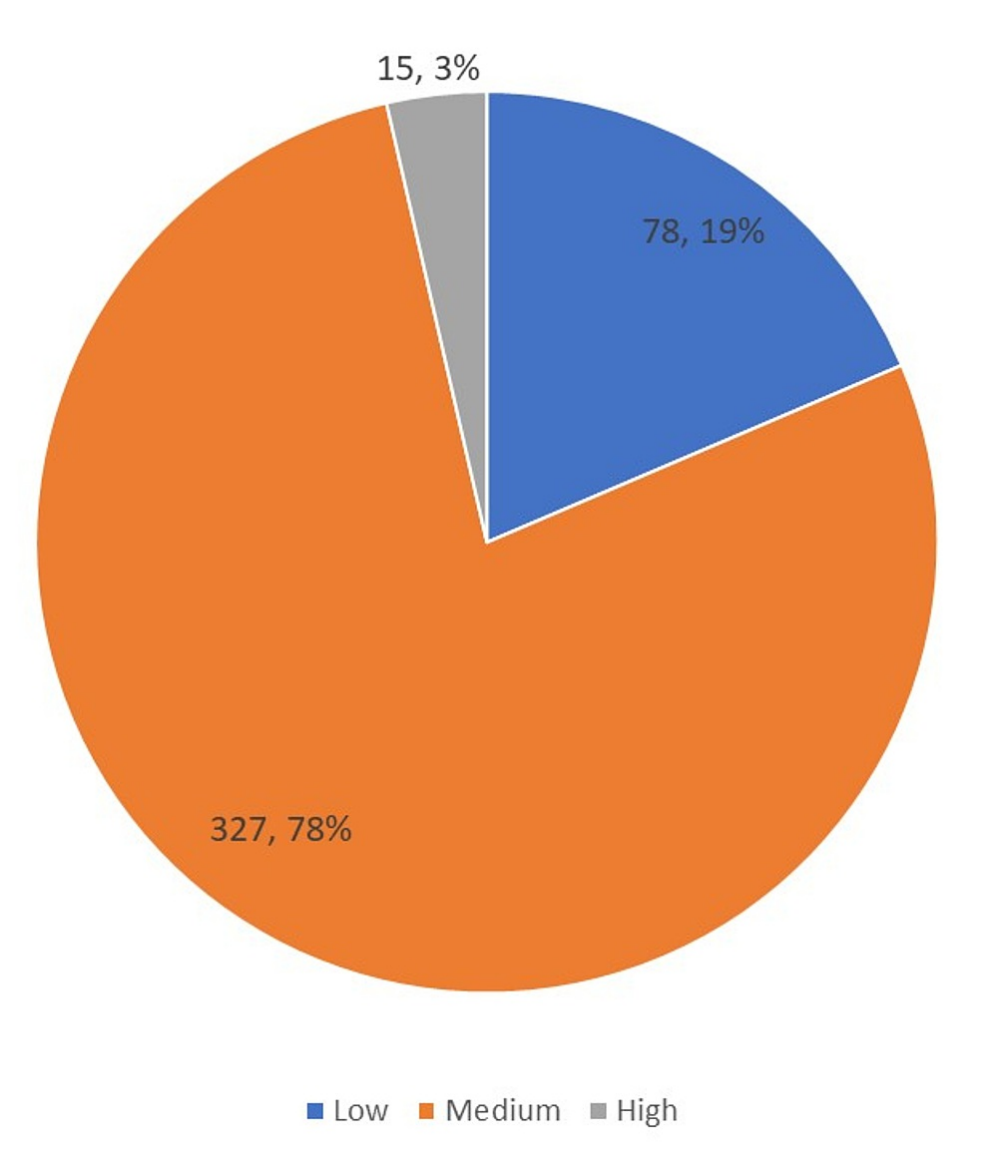
Perceived stress among mothers in Arar, KSA (n = 420)

Around 78% of the mothers perceived medium stress, 19% perceived low stress, and 3% perceived high stress (Figure 2).

On multivariate analysis by ordinal logistic regression, only the type of delivery (adjusted odds ratio (OR): 2.42; 95% confidence interval (CI): 1.30-4.52), suffering from anemia (adjusted OR: 2.81; 95% CI: 1.43-5.52), mothers’ education (adjusted OR: 1.35; 95% CI: 0.73-2.47), and NE among the youngest child (adjusted OR: 2.14; 95% CI: 1.05-4.37) were statistically significant factors associated with a higher level of stress (Table 3).

**Table 3:** Multivariate ordinal logistic regression analysis of the association between nocturnal enuresis and perceived stress among mothers in Arar, KSA *P-value < 0.05

Variables	Adjusted OR (95% CI)	P-value
Age of the youngest child (years)	1.09 (0.96–1.23)	0.181
Type of delivery		
Vaginal	2.42 (1.30–4.52)	0.005*
Cesarean section		
Hospital admission after delivery		
No	0.56 (0.28–1.09)	0.087
Yes		
Sibling suffering		
No		
Yes	0.61 (0.23–1.61)	0.32
Suffering from anemia		
No	2.81 (1.43–5.52)	0.003*
Yes		
Suffering from diabetes mellitus		
No	2.12 (0.60–7.48)	0.24
Yes		
Suffering from psychological problems		
No	2.04 (0.79–5.22)	0.139
Yes		
Mother’s education		
Primary/preparatory	0.48 (0.24–0.96)	0.039*
Secondary	1.35 (0.73–2.47)	0.336
University or more		
Family income (perceived)		
Enough	0.85 (0.34–2.15)	0.734
Not enough	2.58 (1.36–4.88)	0.004*
Enough and more		
Youngest child has nocturnal enuresis		
No		
Yes	2.14 (1.05–4.37)	0.036*

## Discussion

This study aimed to assess the burden of NE and its association with the mental health of mothers. We found that the prevalence of NE was 23.9%. This prevalence is higher as compared to most other studies in KSA. In the Taif region, the frequency of nocturnal enuresis was 7.81% [14] and 31.2% in a study in different regions of the kingdom [15]; in Riyadh, the prevalence of NE was 18.5%, with a higher burden among male children [16]. In Jazan, 76.4% of the children had NE [17], the prevalence of nocturnal enuresis in Hail was 22.7% [18], and it was 92.8% in Jeddah [21]. Compared to our findings, the prevalence of NE was 18% in Egypt [19] and 6.8% in Iran [26]. The disparity in the prevalence could be due to the different definitions used in different studies and the differences in the study participants and sampling. Furthermore, these differences can also be attributed to varying underreported behaviors in different settings as this condition may be stigmatized and therefore parents may not report NE in their children.

We found that 11% of enuretic children had daytime and nighttime symptoms, which is in line with another study in which 55% of children had these symptoms [15]. On the other hand, one study by Sarici et al. reported a lower prevalence of 18% of daytime symptoms [27].

In our study, there was a significant association between NE and a higher level of stress. Multivariate analysis showed that the type of delivery, history of anemia, mothers’ education, and NE among the youngest children were significantly associated with perceived stress. A number of factors have been associated with NE, which include the child’s age and gender, a family history of NE, pinworm infestation, lack of breastfeeding, poor academic performance, the level of parents’ education, socioeconomic status, deep sleep, a history of urinary tract infections, a history of hospital admission after delivery, a history of diabetes, daytime enuresis, and tea and sweets consumption [14,15,17-21,28].

Enuresis results from the interaction of multiple physical and psychological factors. Our study participants perceived that enuresis may be caused by weakness in the muscles of the lower urinary tract (23%), urinary tract infections (21.1%), and psychological problems (18.9%). In a study, according to the parents, the most common causes of enuresis is deep sleep (56%), unknown (39%), hesitancy to go to the washroom (26%), and lower bladder capacity (21%) [19]. These perceptions are persistent for a long time and did not change.

Improvement of the condition was observed in 76% of the sample on decreasing fluid intake before sleeping. Sherah et al. reported that most parents relied mainly on fluid restriction and emptying the bladder before sleeping to manage nocturnal enuresis [17]. This finding has practical implications in that advising parents of children with NE could help reduce the enuresis and thus its effects on the child and parents.

Primary nocturnal enuresis is a demanding clinical condition that is difficult to control and significantly impacts family life. It is also assumed that a new enuretic crisis, as an uncontrollable event, becomes a stressful event that adds on to the previous ones with a cumulative effect, as confirmed by Nederhof and Schmidt (2012) [29]. We found that NE was associated with a higher risk of being stressed, among mothers. Of the participants, 78% had medium perceived stress, 19% had low perceived stress, and 3% had high perceived stress. This finding is consistent with the study of De Bruyne et al. (2009) that relates primary nocturnal enuresis to parenting stress [30]. These findings indicate that NE is a stressful event for parents and can harm overall family life.

This study is one of its kind to assess the burden of NE in Arar and see its association with the mental health of mothers in Saudi Arabia. We used a validated tool for the assessment of perceived stress. However, certain limitations should be considered while interpreting the results of this study. For starters, the sample was only powered for NE prevalence, which may not be sufficient for the associations observed in this study. The participants were also recruited conveniently from one city only, so the generalizability of the results may be limited.

## Conclusions

In conclusion, the prevalence of nocturnal enuresis in Arar is among the previously reported figures in Saudi Arabia and worldwide. There was a significant association between NE among the youngest children and a higher level of stress. Other factors associated with stress were the type of delivery, suffering from anemia, and mothers’ educational level. Governmental and nongovernmental organizations should work together to organize workshops and interventions to raise parental and community awareness about NE, its psychological effects, and its treatment options.

## References

[1] Kiddoo DA (2012). Nocturnal enuresis. CMAJ.

[2] Stein MA, Mendelsohn J, Obermeyer WH, Amromin J, Benca R (2001). Sleep and behavior problems in school-aged children. Pediatrics.

[3] Sadock BJ, Sadock VA (2015). Kaplan & Sadock’s comprehensive textbook of psychiatry, 9th edition.

[4] Shreeram S, He JP, Kalaydjian A, Brothers S, Merikangas KR (2009). Prevalence of enuresis and its association with attention-deficit/hyperactivity disorder among U.S. children: results from a nationally representative study. J Am Acad Child Adolesc Psychiatry.

[5] Thiedke CC (2003). Nocturnal enuresis. Am Fam Physician.

[6] Nørgaard JP, Djurhuus JC, Watanabe H, Stenberg A, Lettgen B (1997). Experience and current status of research into the pathophysiology of nocturnal enuresis. Br J Urol.

[7] Equit M, Klein AM, Braun-Bither K, Gräber S, von Gontard A (2014). Elimination disorders and anxious-depressed symptoms in preschool children: a population-based study. Eur Child Adolesc Psychiatry.

[8] Van Hoecke E, Baeyens D, Vande Walle J, Hoebeke P, Roeyers H (2003). Socioeconomic status as a common factor underlying the association between enuresis and psychopathology. J Dev Behav Pediatr.

[9] Biederman J, Santangelo SL, Faraone SV (1995). Clinical correlates of enuresis in ADHD and non-ADHD children. J Child Psychol Psychiatry.

[10] Hägglöf B, Andrén O, Bergström E, Marklund L, Wendelius M (1997). Self-esteem before and after treatment in children with nocturnal enuresis and urinary incontinence. Scand J Urol Nephrol Suppl.

[11] Butler RJ (1998). Night wetting in children: psychological aspects. J Child Psychol Psychiatry.

[12] El-Defrawi MH, Amin SE, Zeitoun AE, Ragab HA (1997). Social competencies, behavioral, psychological and cognitive correlates in children with nocturnal enuresis. Egypt J Psychiat.

[13] Ghotbi N, Kheirabadi GH (2001). Prevalence of nocturia and its associated factors in primary school children in Sanandaj in 2002. J Kurdistan Univ Med Sci.

[14] Al-Zahrani SS (2014). Nocturnal enuresis and its treatment among primary-school children in Taif, KSA. Int J Res Med Sci.

[15] Alhifthy EH, Habib L, Abu Al-Makarem A (2020). Prevalence of nocturnal enuresis among Saudi children population. Cureus.

[16] Alshahrani A, Selim M, Abbas M (2018). Prevalence of nocturnal enuresis among children in Primary Health Care Centers of Family and Community Medicine, PSMMC, Riyadh City, KSA. J Family Med Prim Care.

[17] Sherah KM, Elsharief MW, Barkat NA, Jafery AM (2019). Prevalence of nocturnal enuresis in school-age children in Saudi Arabia. Int J Med Develop Ctries.

[18] Shahin MM, Al-Shamary YW, Ahrashed LK, Al-Motiri SS, Alhazmi SM, Alswab WS (2017). The epidemiology and factors associated with nocturnal enuresis among school & preschool children in hail city, saudi arabia: a cross-sectional study. Int J Adv Res.

[19] Hamed A, Yousf F, Hussein MM (2017). Prevalence of nocturnal enuresis and related risk factors in school-age children in Egypt: an epidemiological study. World J Urol.

[20] Schlomer B, Rodriguez E, Weiss D, Copp H (2013). Parental beliefs about nocturnal enuresis causes, treatments, and the need to seek professional medical care. J Pediatr Urol.

[21] Ashoor SJ, Alghalbi DS, Alsubhi HA, Al Herz FA (2021). Pediatric nocturnal enuresis prevalence in association with multiple risk factors: a cross-sectional study in Jeddah, KSA. Indo Am J Pharm Sci.

[22] Shaheen DG, El-Masry R, Hammad A, Montasser N (2021). Nocturnal enuresis and its effect on quality of life among Egyptian children. Ann Pediatr.

[23] Cohen S, Kamarck T, Mermelstein R (1994). Perceived stress scale. Measuring stress: a guide for health and social scientists.

[24] Chaaya M, Osman H, Naassan G, Mahfoud Z (2010). Validation of the Arabic version of the Cohen Perceived Stress Scale (PSS-10) among pregnant and postpartum women. BMC Psychiatry.

[25] Ali AM, Hendawy AO, Ahmad O, Al Sabbah H, Smail L, Kunugi H (2021). The Arabic version of the Cohen Perceived Stress Scale: factorial validity and measurement invariance. Brain Sci.

[26] Safarinejad MR (2007). Prevalence of nocturnal enuresis, risk factors, associated familial factors and urinary pathology among school children in Iran. J Pediatr Urol.

[27] Sarici H, Telli O, Ozgur BC, Demirbas A, Ozgur S, Karagoz MA (2016). Prevalence of nocturnal enuresis and its influence on quality of life in school-aged children. J Pediatr Urol.

[28] Yousef KA, Basaleem HO, bin Yahiya MT (2011). Epidemiology of nocturnal enuresis in basic schoolchildren in Aden Governorate, Yemen. Saudi J Kidney Dis Transpl.

[29] Nederhof E, Schmidt MV (2012). Mismatch or cumulative stress: toward an integrated hypothesis of programming effects. Physiol Behav.

[30] De Bruyne E, Van Hoecke E, Van Gompel K, Verbeken S, Baeyens D, Hoebeke P, Vande Walle J (2009). Problem behavior, parental stress and enuresis. J Urol.

